# Relative Contribution of Each Component of the French Ante-Mortem Surveillance System for Bovine Tuberculosis in Its Overall Sensitivity

**DOI:** 10.3390/microorganisms9030643

**Published:** 2021-03-19

**Authors:** Valentine Guétin-Poirier, Julie Rivière, Barbara Dufour

**Affiliations:** Anses, Ecole Nationale Vétérinaire d’Alfort, Laboratoire de Santé Animale, USC EPIMAI, F-94700 Maisons-Alfort, France; julie.riviere@vet-alfort.fr (J.R.); barbara.dufour@vet-alfort.fr (B.D.)

**Keywords:** bovine tuberculosis, scenario tree modeling, surveillance, sensitivity

## Abstract

The aim of this study was to assess the contribution to the sensitivity of the French ante-mortem surveillance system for bovine tuberculosis in cattle of each of the system’s components (periodic screening, epidemiological investigations, and screening exchanged animals), on a local scale defined by administrative areas. These components were individually assessed in previous studies by scenario tree modeling. We used scenario tree modeling at the herd level and combined the results to evaluate the overall sensitivity of the ante-mortem surveillance system. The probability to detect at least one infected herd was consistent with the location of the outbreaks detected in 2016. In areas with a high apparent incidence, the probability of an infected herd to be detected was satisfactory (for an infected herd there was a 100% probability to be detected over a two-year period). Periodic screening was the most important component for the overall sensitivity in infected areas. In other areas, where periodic screening had stopped, tracing-on epidemiological investigation was the most sensitive component of the system. Screening exchanged animals had a negligible part in the overall sensitivity of the surveillance system.

## 1. Introduction

Bovine tuberculosis (TB) is an infection caused by *Mycobacterium bovis*, *M. tuberculosis*, or *M. caprae*. TB is a chronic infection that is mostly asymptomatic in cattle. In developed countries, with the reduction of TB prevalence in cattle and milk pasteurization, TB is no longer a public health issue [1,2,3] but an economical one. Indeed, officially the TB-free status is important to facilitate livestock exchange between countries within the European Union. Since 2001, France is officially TB-free, meaning that its incidence is lower than 0.1%. Nevertheless, despite 70 years of TB control measures, TB eradication is still not achieved and TB incidences are increasing in the South-Western areas, putting the TB-free status of France in jeopardy. In this context, the evaluation of the French TB surveillance system in cattle was essential for the identification of improvement leverage.

In France, TB surveillance in cattle relies on many components: the post-mortem surveillance system (looking for TB-like lesion on every culled animal) and the ante-mortem surveillance system composed by periodic screening with the intradermal cervical tuberculin test (ICT), epidemiological investigations (tracing-on and tracing-back investigations), and screening of exchanged animals with ICT. We studied the ante-mortem surveillance system.

For periodic screening, three mandatory protocols can be used. They all begin with the screening of animals older than 24 months in the herd with ICT (single ICT (SICT) or comparative ICT (ICCT) depending on the area). If at least one of the cattle has a non-negative result with this ICT, the herd is considered as “suspect” of TB and investigations follow. In the “strict” protocol, the animals with non-negative results are culled to perform laboratory analyses (histology, PCR, and culture); if TB is not detected, the herd will be screened again six weeks later with ICCT. In the “compliant quick path” protocol, animals with non-negative results are culled and the results of the laboratory analyses performed define the status (infected or uninfected) of the herd, without a second screening with ICCT in the herd. In the “compliant slow path” protocol, non-negative animals are tested with the interferon gamma test (IFN): if they obtain a positive result, they are culled for laboratory analyses; if they are not, they are screened a second time with ICCT. 

In herds found to be linked with an outbreak by tracing-back investigations (upstream link), the “strict” protocol of periodic screening is used. Downstream link herds (from tracing-on investigations) can be investigated with various protocols, depending on the linked animal (if it is still present in the herd or not). If the linked animal is no longer in the herd, the strict protocol of periodic screening is used. If the linked animal is still in the herd, two protocols can be used: the “trace and cull” protocol leading to the systematic culling of this animal, whereas in the “trace and test” protocol the linked animal is culled only if it obtained a non-negative result with an ICCT. 

The screening of exchanged animals is performed with SICT or ICCT. It is applied when the time between the departure of the selling herd and the arrival of an animal at the buyer’s herd exceeds six days, or when the animal comes from a herd classified as “at risk”.

Each component of the TB ante-mortem surveillance system was evaluated separately in previous studies [4,5,6]. In this study, the ante-mortem surveillance system, as a whole, was evaluated at the administrative areas’ level. The objective was to estimate its sensitivity in each area and to identify the component(s) contributing the most to the surveillance system’s sensitivity.

## 2. Materials and Methods

A scenario tree was used with a stochastic modeling approach allowing to account for the uncertainty and the variability of the parameters. In this method, a scenario tree describing each possible pathway of the studied surveillance system is developed. Factors influencing the probability of infection or the probability of detection must be introduced into the tree as category nodes. From each node, one issues as many branches as there are categories. The infection node has two branches: infected/uninfected. Detection nodes are mostly test results. Once the tree is complete, it must be implemented: a probability of occurrence must be attributed to each branch of the tree. The probability of occurrence of each pathway is calculated by multiplying the probability of occurrence of each of its branches. Finally, the global sensitivity of the system can be calculated by adding the probability of occurrence of all pathways leading to the detection of the infection. 

**Figure 1:**
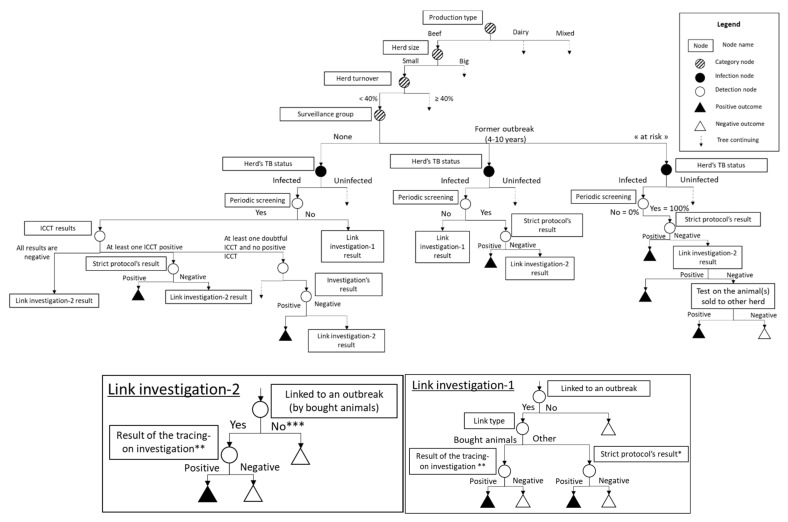
Scenario tree used to model the French *ante-mortem* TB surveillance and its various components. * Strict protocol = protocol to investigate TB suspicions following periodic screening with ICCT [4,6]. ** “trace and cull” protocol = tracing-on investigation protocol in which linked animals (= bovines bought from the outbreak) are systematically culled [5]. *** The herd is either not linked to any outbreak or linked by neighboring or by selling animals to the outbreak. In this last case, no further investigations are led because the herd had already been screened during periodic screening.

Previously constructed trees for each of the ante-mortem surveillance components were re-used and combined into an overall tree presented in Figure 1. The scenario tree was set up from published studies, experts’ opinion (in particular the national coordinator of TB surveillance), and/or field data. The field data were extracted mainly from the SIGAL database which centralized the results of TB surveillance and control at the herd level, and the BDNI database that registers birth and death dates, as well as herds identification and movements of all bovine living in French herds.

### 2.1. Category Nodes

The category nodes characterizing the type of herd (production type, size, herd turnover) have been retained because of their impact on the probability of infection and/or of detection. The production types could be dairy, beef, or mixed (dairy and beef). The herd size was defined depending on the production type as small or big. The limit defining these two categories was the mean number of females older than 24 months of the concerned production type (74 for dairy, 66 for beef, and 124 for mixed herds, according to the BDNI database). The turnover was defined as the number of animals introduced in the herd within the year (excluding births) divided by the mean number of animals in the herd within the year. Two herd turnover categories were defined: low and high turnover, with a limit at 40% (mandatory limit in France from which herds have additional constraints regarding TB surveillance).

A category node “surveillance group” was added. This node corresponded to the classification of the herd by animal health authorities as a former outbreak or as a herd “at risk”. This classification influences the screening protocol used and the surveillance components applied in the herd. Indeed, in the event of suspicion following periodic screening, a former outbreak (up to 10 years ago) or a herd classified as “at risk” is systematically investigated using the “strict” protocol. In addition, an annual screening is carried out in herds classified as “at risk” (whereas in other herds the screening could be spaced out) and the animals sold by this herd type are screened with ICCT.

### 2.2. Likelihood for a Herd to Be Classified “at Risk”

Although the animal health authorities at the national level clearly define the situations in which herds must be classified as “at risk”, it appears field practices differ depending on the administrative area (personal communications of the national TB coordinator and animal health director of health authorities’ services). Unfortunately, there is no centralized exhaustive list of herds classified as “at risk” that would have made it possible to easily estimate the probability of a herd being classified as “at risk” in each area.

On the field, the following herds are generally classified as “at risk”: former outbreaks less than three years ago (inclusive) and herds linked with an outbreak by the purchase of an animal (downstream linked herd), in which the infection was not detected and which have kept the linked animal (“trace and test” protocol) (personal communication of the national TB coordinator). The other types of situations in which a herd is considered as “at risk” are evaluated on a case-by-case basis and were not included in our model. In some areas, herds with a high turnover rate are classified as “at risk” but are only screened for introduced animals in order to protect the herd from the possible introduction of TB. We therefore excluded them from the “at risk” classification. The probability for a herd of type t (defined by the type of production, the size, and the turnover of the herd) of a specific area to be classified as “at risk” was estimated by summing the probability for such a herd to have had a former outbreak (1–3 years) (determined from SIGAL database for the year 2016) with the product of: (i) the probabilities for the same herd to be a downstream linked herd (cf. corresponding paragraph); (ii) the probabilities for the same herd to be investigated by the “trace and test” protocol (probability estimated at 0.05 by experts); and (iii) the probability that the linked animal is still present in the herd and that it has obtained a negative result on ICCT test.

### 2.3. Probability for the Herd to Be a Former Outbreak Older Than 3 Years

For each area and for each type of herd, the probability that a former outbreak in the herd was more than three years ago was modeled by a fixed probability corresponding to the proportion existing in 2016 in each area (SIGAL database).

### 2.4. Adjusted Relative Risks of Infection

For each of the factors identified as affecting the probability of a herd being infected (herd size and herd turnover, herd surveillance group: “at risk” or previous outbreak older than three years), an adjusted relative risk of infection was estimated to account for their higher probability of infection. First, we modeled multiplier coefficients reflecting the over-risk of infection caused by each of these factors. 

Many studies have shown a statistical association between the herd size and the probability of a herd becoming infected [7,8,9,10,11,12,13,14]. Most of these studies estimated ORs representing the increased risk for a herd to be infected when increasing the herd size by one log unit [9,10,11] or when comparing size classes with very small herds [12], which was not easily incorporated into our model based on a small/large herd binary distinction. We therefore used only the results of the study that compared herd in a binary manner (large vs. small) [13]. We thus modeled the multiplier coefficient reflecting the over-risk of infection in large herds (C_big_) as an asymmetric normal distribution N_asym_ (mean = 2; CI95% min limit = 1.4; CI95% max limit = 3).

Regarding the influence of herd turnover, most studies focused on the purchase of animals from an infected market or herd [11,14] and obtained ORs of 1.95 [1.05–3.63]_95% CI_ and 2.6 [1.20–5.63]_95% CI_, respectively. However, one study looked at the number of animals purchased regardless of their origin and showed that purchasing more than 27 animals per year increased the probability of a herd being infected (OR = 1.23 [1.03–1.49]_95% CI_) [15]. This number of 27 animals purchased per year corresponds to a turnover higher than 40% for all herds with less than 68 animals. This was fairly close to the average herd size of beef and dairy herds in our model. We therefore used the results of this publication to model the multiplier coefficient reflecting the impact of a high turnover on the probability of a herd being infected (C_high turnover_), using rounded values to account for the uncertainty in this coefficient. Thus, C_high turnover_ was modeled by an asymmetric normal law N_asym_ (mean = 1.2; CI95% min limit = 1; CI95% max limit = 1.5).

Finally, some studies highlighted the TB history of the herd as a risk factor for infection, notably in England [12] and Belgium [16,17] (coefficient estimated at 2). Similarly, a French study had previously demonstrated an over-risk of infection (OR of approximately 80) in former outbreaks cleared from infection by selective culling (compared to those cleared by total culling) [18]. However, this study had been conducted using old data from selective culling protocols that were different from those used at the time of our study and were less effective. Nevertheless, given the capacity of resistance of the mycobacterium in the environment, it seemed reasonable to assume that former breakdowns in France had an over-risk of TB infection. From the SIGAL data available for the years 2014 to 2016, we estimated an over-risk of infection coefficient for former outbreaks (C_risky_) by grouping herds either by areas (average coefficient of 1.8) or by herd type (average coefficient of 3). The coefficients varied each year and the amount of data available did not allow us to highlight significant differences between herd types or areas. We therefore modeled this coefficient, regardless of the area and the herd type, using a Pert distribution law with a minimum value of 1 (absence of over-risk of being infected), a most probable value of 2, and a maximum value of 10 (maximum coefficient observed in the areas), to represent the diversity of the possible situations.

As data on herds classified as “at risk” were not available, and as the “at risk” herds included former outbreaks less than three years old, we assumed that the “at risk” herds had an over-risk of being infected of the same order as that of former TB outbreaks. We therefore used the same coefficient (C_risky_) to model the influence of the “at risk” classification on the probability of a herd being infected with TB.

In the model, the relative risk of TB infection (RRi) for each herd category defined by size, turnover, and surveillance group, was estimated as shown in Table 1.

Finally, for each herd category, the adjusted relative risk (Ar_i_) was calculated according to Equation (1) so that the sum of the twelve calculated adjusted relative risks was equal to one.

(1) $${\mathrm{Ar}}_{i}=\frac{\mathrm{RRi}}{\sum_{j=1}^{k}\mathrm{RRj}\times \mathrm{PrPi}}$$

where k is the number of branches, PrPi the proportion of herds belonging to the i category, i being one of the herd categories defined by the size, the turnover, and surveillance group of the herd.

### 2.5. Probability of Infection

In a first approach, the areas were classified into three groups according to the number of outbreaks detected between 2011 and 2016 (0–5 outbreaks, 5–10 outbreaks, and more than 10 outbreaks). The prevalence in each group was set at 0.01%, 0.02%, and 0.6%, respectively, which corresponds to the average apparent incidence in 2016 in each group (SIGAL database). Details by areas are provided in Appendix A, Table A1. Subsequently, a further evaluation was carried out by setting the prevalence at 0.1% (the European threshold for maintaining TB-free status) for all areas, in order to compare the effectiveness of ante-mortem surveillance at equal prevalence. 

The residual probability of infection of a herd was determined in each branch of the model by multiplying the prevalence with the adjusted relative risk corresponding to the category of the herd.

Intra-herd prevalence was modeled like in the previous studies [4,5] by a Pert distribution law (Pert (min = 0.008, mode = 0.0094, max = 0.031)) based on knowledge of intra-herd prevalence in French herds.

### 2.6. Periodic Screening on Herds

#### 2.6.1. Likelihood of a Herd Being Subject to Periodic Screening

For herds not classified as “at risk”, the probability of being subjected to periodic screening was modeled, for each area, by a value set according to field data. Indeed, we used the number of herds actually screened for the 2016–2017 screening campaign (SIGAL database) and the number of existing herds in the department on 1 January 2016 (extracted from the BDNI database). For herds classified as “at risk”, which are screened annually, this probability was set to one.

#### 2.6.2. Periodic Screening Protocol Used

In herds classified as “at risk” and in former outbreaks, the “strict” protocol is systematically used. In other herds, the protocol used is conditioned by the results obtained to the first ICCT screening (ICCT1). Therefore, an additional detection category node, entitled “ICCT results”, which corresponds to the ICCT1 results of the herd, has been added to the tree. Three branches are derived from this node: (i) “At least one ICCT1 positive “, which is continued with the “strict” protocol; (ii) “All results are negative”, which is continued with no further investigations; and (iii) “At least one doubtful ICCT but no positive ICCT”, which is continued with either the “compliant quick path” or “compliant slow path” protocol (Figure 1).

The probability of a herd of size N obtaining only negative results in the periodic screening was calculated based on the sensitivity and specificity of the ICCT (modeled as presented in a previous study [6] (respectively, N_asym_ (mean = 0.74; CI95% min = 0.43; CI95% max = 0.95) and N_asym_ (0.99; 0.80; 100), and according to Equations (2) and (3) for an infected herd (I) and an uninfected herd (NI), respectively:

(2) $$p\;\left(\mathrm{all}\;\mathrm{negative}\;│\;I\right)\;=\;{\left(1\;-{\;\mathrm{Se}}_{\mathrm{ICCT}}\right)}^{N\;\times \;\mathrm{pr}\_\mathrm{intra}-\mathrm{herd}}\;\times {\;\mathrm{Sp}}_{\mathrm{ICCT}}{}^{N\;\times \;\left(1\;-\;\mathrm{pr}\_\mathrm{intra}-\mathrm{herd}\right)}$$

(3) $$p\;(\mathrm{all}\;\mathrm{negative}\;|\;\mathrm{NI})\;={\;\mathrm{Sp}}_{\mathrm{ICCT}}{}^{N}$$

For a herd of size N, the probability of obtaining at least one positive ICCT at periodic screening was calculated according to Equations (4) and (5) for an infected herd (I) and an uninfected herd (NI), respectively:

(4) $$\begin{array}{cc} p\;(\geq \;1\;\mathrm{ICCT}\;+\;|I) & \\ & =\;1-\;{\left(1\;-{\;\mathrm{Raw}\;\mathrm{Se}}_{\mathrm{ICCT}}\right)}^{N\;\times \;\mathrm{pr}\_\mathrm{intra}-\mathrm{herd}}\\ & \times {\;\mathrm{Raw}\;\mathrm{Sp}}_{\mathrm{ICCT}}{}^{N\;\times \;\left(1-\mathrm{pr}\_\mathrm{intra}-\mathrm{herd}\right)} \end{array}$$

(5) $$p\;(\geq \;1\;\mathrm{ICCT}\;+\;|\mathrm{NI})\;=\;1\;-{\;\mathrm{Raw}\;\mathrm{Sp}}_{\mathrm{ICCT}}{}^{N}$$

Raw Se_ICCT_ and raw Sp_ICCT_ are the sensitivity and specificity of the test when doubtful results are considered negative, whose modeling (raw Se_ICCT_ = Se_ICCT_ × 0.6 and raw Sp_ICCT_ = 1 − [(1 − Sp_ICCT_) × 0.15]) has been deduced from French data as detailed in calculations A1.

The probability of a herd obtaining at least one non-negative ICCT but no positive ICCT was the complementary to one of the two previous probabilities.

When at least one ICCT is non-negative but none is positive, the protocol applied can be either the “compliant quick path” or the “compliant slow path” protocol. The choice of protocol is then made on a case-by-case basis by decision of the health authorities. We thus estimated, from the SIGAL data on the screening campaign 2015–2016, that in 37 areas, the “compliant quick path” protocol was systematically used, whereas it was less frequently applied in areas 13 (11%), 2B (7%), 21 (7%), 66 (0%), and 87 (79%). For the areas in which we did not have enough data (54 areas), the probability of using the “compliant quick path” protocol was modeled by a Pert distribution law, with parameters set according to expert opinion (national TB coordinator): Pert (min = 0.2; mode = 0.4; max = 0.6).

#### 2.6.3. Sensitivity and Specificity of Protocols

In the model, the probabilities of obtaining a positive or negative result for the “strict” protocol, used in the “former outbreak” and “at risk” categories, were taking into account the adaptations of ICCT practices by the veterinarian, as described in a previous study [6]. 

For other herds, these probabilities were estimated conditionally of having at least one positive result on the ICCT1 for the “strict” protocol and conditionally of having at least one non-negative result on the ICCT1 but no positive result for the “compliant quick path” and “compliant slow path” protocols according to the method described in calculations A2.

### 2.7. Epidemiological Investigations

#### 2.7.1. Likelihood of a Herd Having a Downstream Link to a TB Outbreak

For each area, we randomly sampled 2000 herds of each production type in the BDNI. For each of these herds, we estimated their size on 1 January 2016 and their turnover rate on the same date, in order to have a sample of herds for each herd type in each area. For each herd, the list of animals purchased between 1 January 2010 and 1 January 2016 (i.e., over a five-year period) and the identification numbers of the herds of origin of these animals as well as their size, production type, turnover rate, and administrative area were extracted from the BDNI. Thus, for each herd in the sample, the herds that could potentially cause a downstream link in 2016 were identified (“seller herds”). The probability for each of these herds to experience an outbreak in 2016 was estimated by multiplying the prevalence set in the model for the area to which they belonged (see Section 2.5) by the adjusted relative risk associated with the type of herd (see Section 2.4). Then, for each of the sampled herds, the probability that at least one of its seller herds will experience an outbreak in 2016—thus corresponding to the probability for the sample herd to be a downstream link to a TB outbreak—(p_downstreamlink) was estimated using Equation (6), where p_outbreak_k_ was the probability for the kth vendor herd to experience an outbreak in 2016 and k was the number of vendor herds:

(6) $$p\_\mathrm{downstreamlink}\;=\;1\;-\;\prod_{k}(1\;-\;p\_{\mathrm{outbreak}}_{k})$$

**Figure 2:**
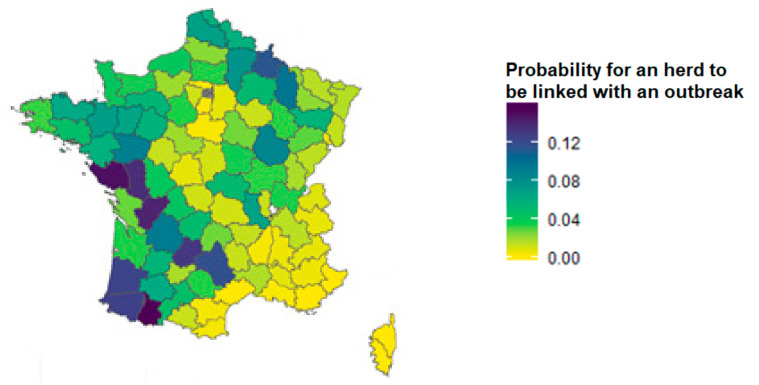
Mean probability of a herd having a downstream epidemiological link to a TB outbreak used in the scenario tree.

Thus, for each herd type in each area, the minimum, maximum, and average values of the probability for a herd to be a downstream link in 2016 were estimated (Figure 2) and were used as parameters for Pert distribution laws to model this probability in the model. When data were missing in an area for a herd type (because the herd type had little or no representation in the area), this probability was set to zero.

#### 2.7.2. Investigation Protocol Used for the Downstream Link

The probability of using the “trace and cull” protocol for the investigation of a downstream link instead of the “trace and test” protocol was set at 95%, based on expert opinion (national TB coordinator). The sensitivity and specificity of these protocols were estimated at each iteration according to the scenario trees presented in a previous study [5].

#### 2.7.3. Likelihood of a Herd Having an Upstream Link to a TB Outbreak

In 2016, 576 epidemiological links were registered in the SIGAL database. From these data, the probability of a herd of a given area and type being epidemiologically linked to a TB outbreak was estimated. If the estimated probability was less than or equal to the probability of the downstream link (Section 2.7.1)—which was the case for 56% of the areas—then the probability for a herd to be linked with a TB outbreak was supposed to be equal to the estimated probability of the downstream link (see Section 2.7.1) (implying the hypothesis of the absence of upstream or neighborhood links). The probability of being implicated in an upstream or neighborhood link was deduced by subtracting the probability of being in a downstream link from the probability of being in a link (all types of links taken together, estimated from the SIGAL database). The investigation of upstream or neighborhood links was modeled according to the “strict” protocol [4,6].

### 2.8. Screening of Exchanged Animals

Screening of exchanged animals is applied when the time between the departure of the selling herd and the arrival of an animal at the buyer’s herd exceeds six days, as well as in herds classified as “at risk”. Exchanges that exceeded six days were not taken into account in our model due to their rarity (personal communication from the national TB coordinator), the lack of data about them, and the very low probability that they could lead to the detection of infection in a herd. Reliable centralized data were not available on the proportion of animals sold from “at risk” herds that are actually screened. Therefore, we assumed that all animals sold from herds classified as “at risk” (with the exception of animals for fattening) were screened. For this herds the proportion of animals sold to another herd was estimated for each herd type of each area thanks to the BDNI data base using 2016 data. The probability of an animal being sold during the year was therefore modeled according to the area and type of herd, using an asymmetrical normal law designed to correspond as closely as possible to the distribution observed in the sample corresponding to the type of herd and the area concerned. 

The probability for each tested animal to be found TB-infected was modeled the same way than for the compliant quick-path protocol of periodic screening (accounting for ICCT sensitivity considering veterinarians’ practices and laboratory analyses results of all animals with non-negative result with the ICCT (histology, PCR, and bacteriology)) [4,6].

### 2.9. Calculations: Likelihood of Detecting Infection in Areas

For each area, the probability of occurrence of each branch of the scenario tree was calculated by multiplying the probabilities of occurrence of the sub-branches composing it. Then, the probability of detecting TB in a herd in the area (p (I ∩ D) herd) was estimated by summing the probability of occurrence of the branches leading to the detection of the infection in an infected herd. This probability was thus estimated for each area, for a prevalence of 0.1% and for a prevalence set according to the group to which the area belonged (see Section 2.5). Finally, the probability of detecting an infection in each area (i.e., the probability of detecting at least one infected herd in the area) was estimated according to Equation (7), where N is the number of herds in the area:

(7) $$\mathrm{probability}\;\mathrm{of}\;\mathrm{detection}\;=\;1\;-\;{\left(1-p\;{\left(I\;\cap \;D\right)}_{\mathrm{herd}}\right)}^{N}$$

In addition, the contribution of each component of the ante-mortem surveillance in the probability of detecting TB infection was estimated for each area.

## 3. Results

### 3.1. Effectiveness of the TB Ante-Mortem Surveillance System

**Figure 3:**
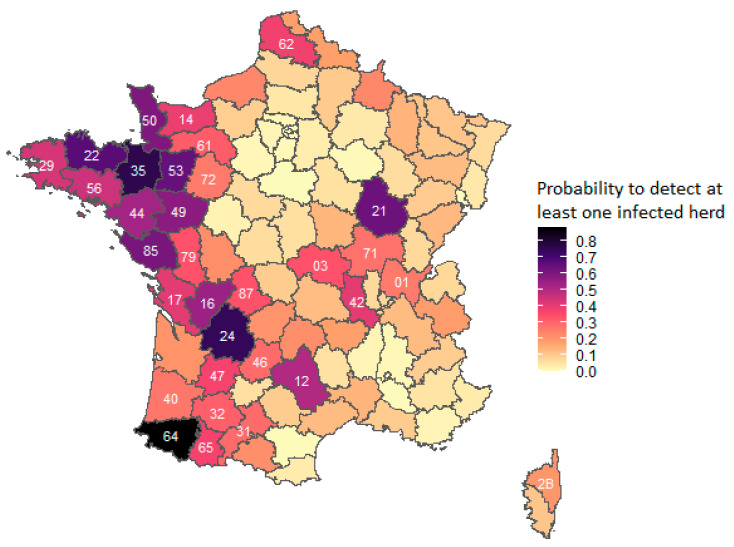
Estimation of the probability of detecting, via ante-mortem surveillance, at least one infected herd in the French administrative areas for a prevalence set at 0.1%. Numbers on the map are the administrative numbers on the areas. For more clarity, only the number of areas mentioned in the text or presented in Figure 4 and Figure 5 are given.

**Figure A1:**
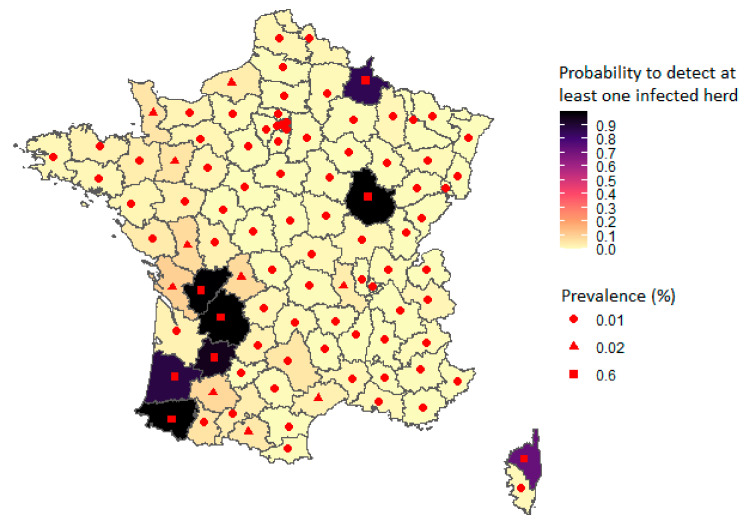
Estimation of the probability of detecting, via ante-mortem surveillance, at least one infected herd in the French administrative areas for a prevalence set according to the number of outbreaks recorded between 2011 and 2016.

The higher the set prevalence, the greater is the likelihood of detecting a TB infection in the area (Figure A1). At equal prevalence (Figure 3), area 64 was the area with the highest probability of detection, followed by area 24. These areas are the ones with the most active TB surveillance (all components of the surveillance system are applied there). The Western areas had a good probability of detecting infection for a fixed prevalence of 0.1%, in contrast to the Central and Southeastern areas.

**Figure A2:**
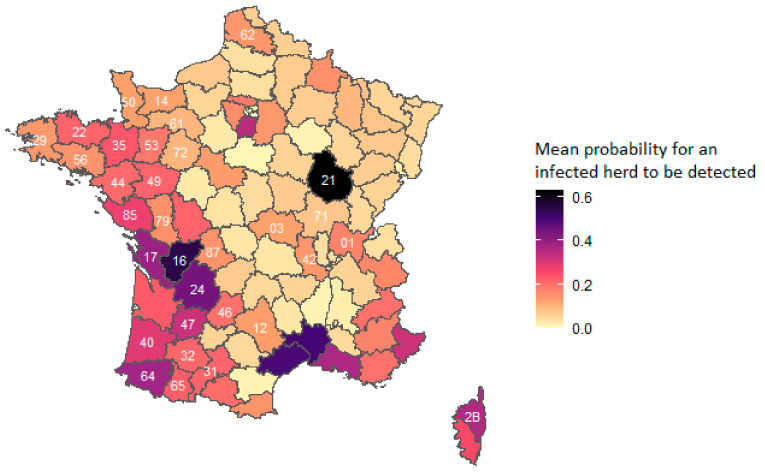
Mean probability for an infected herd to be detected via ante-mortem surveillance for a prevalence set at 0.1%.

**Figure A3:**
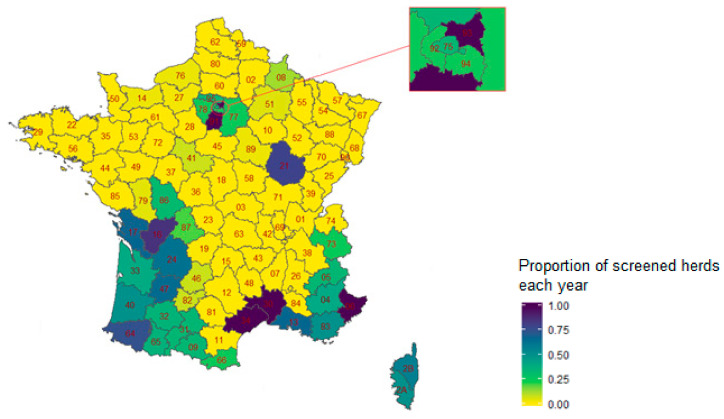
Proportion of herds subject to periodic screening each year as modeled in the scenario tree.

The mean probability for an infected herd to be detected in a given year was highest in areas with high surveillance pressure (periodic screening) (Figure A2 and Figure A3).

### 3.2. Contribution of Each Component in the Overall Sensitivity of the Ante-Mortem Surveillance System

In all areas, the contribution of screening exchanged animals to the overall system sensitivity was negligible (maximum 0.1% of the overall sensitivity).

In the areas, where periodic screening is stopped (50 areas), 100% of the overall sensitivity was ensured by tracing-on investigations, except for two areas (07 and 15), in which tracing-back investigations also contributed to the overall sensitivity. 

**Figure 4:**
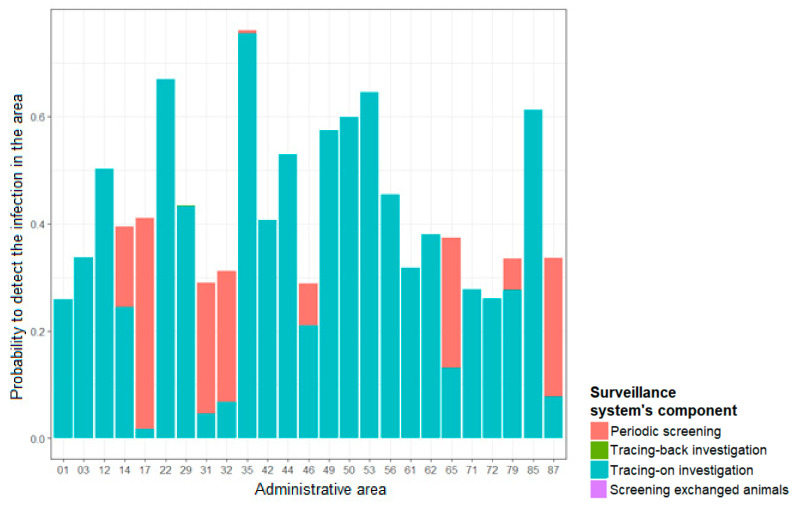
Contribution of each surveillance component in the probability of detecting TB infection, for a prevalence of 0.1% for areas not recently infected and for which this probability is greater than 25%.

In areas without recent infection, tracing-on investigations provided the majority of the sensitivity of the ante-mortem surveillance system, as shown in Figure 4, which is focused on areas where the probability of detecting TB infection was greater than 25%. In areas where periodic screening was maintained in 2016 (areas 17, 31, 32, 46, 65, 79, and 87), this component contributed to a significant proportion of the overall sensitivity of the system. 

**Figure 5:**
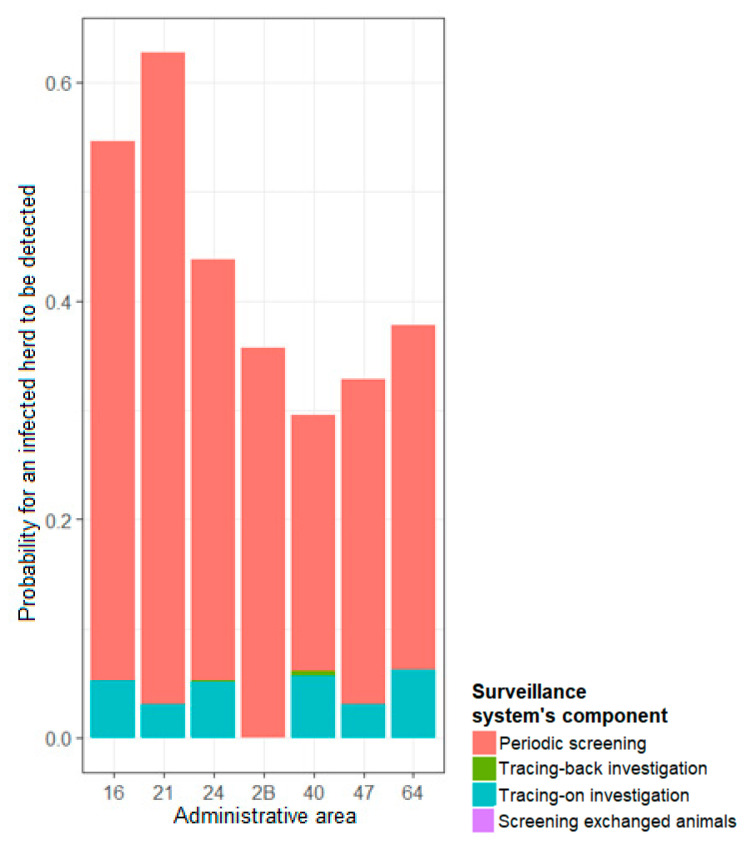
Contribution of each surveillance component in the probability of detecting TB infection for areas with a prevalence of 0.6%.

In areas with a high apparent prevalence (0.6%), periodic screening was the component most likely to detect an infected herd. In those areas, the tracing-on investigations also contributed to a non-negligible proportion of the overall sensitivity of the system (Figure 5).

## 4. Discussion

### 4.1. Material and Method

The probability of a herd being classified as “at risk” was estimated by calculation using the definition of “at risk” herds most commonly used in the field. However, each area seemed to have its own interpretation of the risk classification. It is therefore possible that the number of herds classified at risk was overestimated for some areas and underestimated in others, leading, respectively, to an over- and an under-estimation of the overall sensitivity, in particular of the sensitivity provided by periodic screening.

We assumed that the herds classified as “at risk” had the same over-risk of infection as the former TB outbreaks. This hypothesis can be discussed for outbreaks classified as “at risk” following the investigation of a downstream epidemiological link. Indeed, in these herds, contrary to former outbreaks, TB was never present, and the classification as “at risk” is therefore based solely on the holding of an animal from an outbreak herd that obtained a negative result on ICCT and was not culled. Unfortunately, there are no data to determine the value of the over-risk caused by this epidemiological link compared to previous outbreaks. However, the proportion of herds classified as “at risk” following a downstream link without culling of the linked animal (“trace and test” protocol of the tracing-on investigation) is a priori low because this protocol is rarely chosen (personal communication of the national TB coordinator). The majority of “at risk” herds are therefore former TB outbreaks, which justifies our hypothesis. Former outbreaks classified as “at risk” are more recent (equal or less than three years old) and could therefore have a higher risk of infection than older former outbreaks (4 to 10 years old). Unfortunately, current data and studies under French conditions were insufficient to estimate this possible difference.

The prevalence set in each area to estimate the effectiveness of the system according to the level of infection in the area was based on actual data of the average apparent incidence observed in 2016 in each group of areas. In France, the prevalence and incidence are not exactly equal since some herds may remain outbreaks for up to three years (maximum duration observed for selective slaughter [19]); however, these herds are not concerned by the surveillance measures because infection has already been detected there, and they are therefore in the sanitation phase (total or selective culling).

The probability for a herd to be screened was determined based on the data available in SIGAL and BDNI databases for the 2016–2017 periodic screening campaign. The percentage of herds tested that was registered under SIGAL was consistent with the official screening rates. For some areas, it is possible that some herds registered as subject to periodic screening actually correspond to herds undergoing monitoring (i.e., “at risk” herds), which would lead to an overestimation of the proportion of herds subject to periodic screening and, therefore, of the sensitivity provided by this component of the surveillance system. Furthermore, since this work was carried out, screening rates have changed in some areas, notably by stopping the screening. Our estimates should therefore be updated each time the screening rate changes in an area.

For the protocols used for periodic screening, we have simplified the decision-making scenario. Indeed, the choice of the protocol to be applied is made on a case-by-case basis according to two main criterions: the surveillance category of the herd and the results obtained from the ICCT1. They allow a qualitative estimate of the probability that the suspect herd is actually infected. A strong suspicion (“at risk” or “former outbreak herd and / or positive result on ICCT1) imply the use of the “strict” protocol. A low suspicion implies the use of the “compliant quick path” protocol or the “compliant slow path” protocol). Other criterions (like the presence of infected wildlife in the area) are used for this qualitative estimate, but we did not account for them in our model to avoid its over-complexification. Therefore, it is possible that we slightly underestimated the proportion of strong suspicions and thus the use of the “strict” protocol, which was the most sensitive protocol [4,6], resulting in an underestimation of the overall sensitivity of the system, particularly in the South-West areas, where wildlife is particularly infected.

SIGAL data were used to model the probabilities of using the “compliant quick path” protocol and the “compliant slow path” protocol in cases of low suspicion. We deduced the protocol used from the test results registered in SIGAL for suspect herds in these areas. However, these results are sometimes missing and we were therefore only able to identify the protocols used for 58% of the 1539 suspect herds recorded in SIGAL following the 2015–2016 screening campaign. Thus, the estimated proportions of use of each protocol may have been far from the reality in the field. That may have led, in some areas, to a slight overestimation of sensitivity if the use of the “compliant quick path” protocol was overestimated or an underestimation in the opposite case.

In France, veterinarians’ ICT practices can change from one area to another [20]. We took into account the diversity of veterinarians’ practices but we were unable to do it conditionally to the area [6]. In order to improve the estimation of the overall sensitivity of the system, it would therefore be necessary to carry out more in-depth sociological studies in a few areas of interest. That will allow us collecting more data specific to these areas, which would make it possible to obtain an estimate closer to the field reality and to reduce the uncertainty of the estimates. Our model therefore allows us to compare the effectiveness of the system in each area with supposedly equal veterinarians’ practices, which is not realistic.

We assumed that all animals sold from herds classified as “at risk” (with the exception of animals for fattening) were screened. This assumption is probably optimistic for some areas. Indeed, checking whether an animal comes from an “at risk” herd is currently very tedious via SIGAL (as it requires handling that takes 10 to 15 minutes per animal). In some areas, such as area 21, a person works full time on this verification but this is an exception. The probability of detecting infection through the screening of animals sold by “at risk” herds has therefore been overestimated in most areas.

The probability for a herd to be epidemiologically linked (downstream, upstream, or neighborhood link) was estimated by the herd type and for each area from SIGAL database, but data on the origins of the suspicion were not available for about a quarter of the 1954 suspicions registered under SIGAL during 2016. The number of suspicions due to investigations of epidemiological links was therefore probably underestimated, causing the underestimation of the probability for a herd to be upstream linked or neighborhood linked (as this probability was deduced by subtracting the probability of being downstream linked from the probability of being epidemiologically linked). However, upstream epidemiological links are often investigated during the following year’s periodic screening campaign and were therefore indirectly taken into account in the “periodic screening component” of the model. In addition, neighborhood links only exist in areas with domestic or wild TB outbreaks. In these areas, annual screening is generally in place, at least in the areas around the outbreaks, and a herd already tested in the same year as part of the periodic screening will not be screened again in the event of a neighborhood link. It thus seems consistent that the majority of the epidemiological links investigated are downstream links.

In addition, in some areas, the probability of an epidemiological link (downstream, upstream, and neighbourhood) inferred from SIGAL was lower than the probability of a downstream link estimated from BDNI data and the apparent incidence in each area. This can be explained either by a failure to record in SIGAL database the herds investigated following an epidemiological link, or by a real failure to investigate links in these areas.

To conclude the discussion of the method, Table 2 summarizes the over- and underestimated parameters of the model.

### 4.2. Results

#### 4.2.1. Effectiveness of the Ante-Mortem Surveillance System

**Figure A4:**
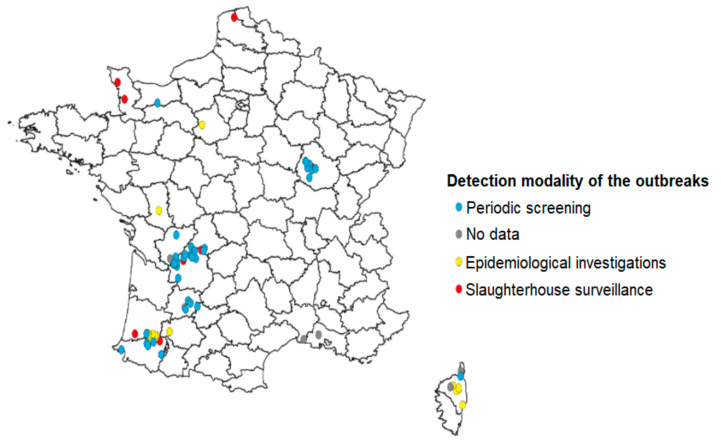
Outbreaks detected in 2016 and component of the surveillance system that detected them.

The probability of detecting at least one infected herd estimated for each area (Figure A1) is consistent with the location of the outbreaks detected in 2016 (Figure A4). Indeed, outbreaks were detected in areas for which the probability of detecting TB was considered high, whereas in areas with a low probability of detection by ante-mortem surveillance, post-mortem surveillance detected outbreaks (slaughterhouse surveillance).

**Figure A5:**
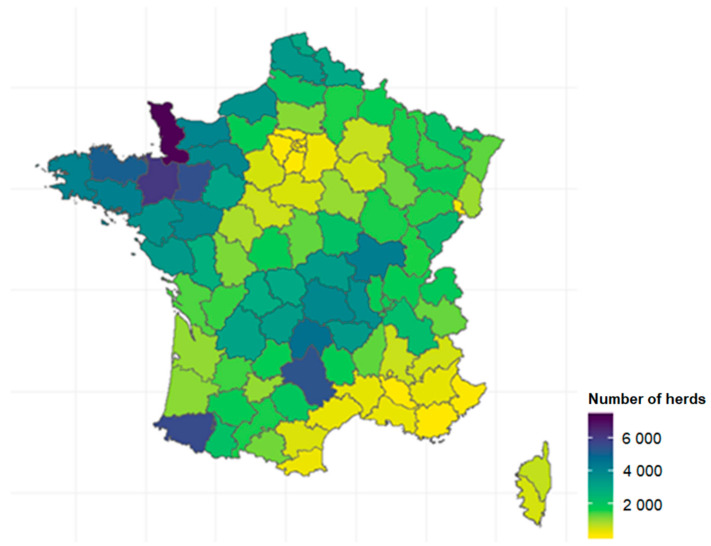
Number of cattle herds registered in the BDNI for the 1 January 2016.

In the absence of periodic screening, the ante-mortem surveillance system provided satisfactory efficiency in areas where there was a significant risk of a herd contamination through a downstream link (Figure 2 and Figure 3). In particular, the areas in the West had a relatively high probability of detecting infection despite a cessation of periodic screening, ensured by the tracing-on investigation component (Figure 4). This can be explained by the fact that, in these areas, there is a large number of herds (Figure A5) and herds had a rather high average probability to be linked to a TB outbreak (Figure 2). In addition, the main production type of the herds of these areas (dairy) ensures a slightly better sensitivity of the tracing-on investigation than for other herd types because of its greater number of animals over 24 months old. This result underlines the importance of the systematic implementation of investigations in the herds identified as a downstream link with a TB outbreak. In other areas without periodic screening, the probability of detecting infection at the 0.1% prevalence limit by ante-mortem surveillance was low; however, post-mortem surveillance improves the overall probability of detection. In addition, if these areas are TB-free (as suggested by the stop of periodic screening) and their herds are unlikely to be linked to a TB outbreak, then the likelihood for them to become infected with the TB agent is very low.

In areas with a high apparent incidence, the probability of an infected herd being detected is satisfactory. In particular, in area 21 the probability of an infected herd being detected was more than 60% over a one-year study period, even though the sensitivity of periodic screening in this area was probably underestimated. Thus, over a two-year period, an infected herd in this area would have a 100% probability of being detected. This result seems consistent with the success of control and surveillance in this area, where the number of outbreaks has been greatly reduced [21]. In the Southwestern areas, the probability of detecting an infected herd was lower but still approached 100% after two to three consecutive years of surveillance.

In high prevalence areas, health authorities establish zones of at least two kilometers around outbreaks. Herds in these zones are considered more likely to be infected and are therefore screened annually. Our study did not take into account this higher probability of infection because of the lack of specific centralized data about these herds. Therefore, in areas practicing this zoning, the surveillance system can be expected to be more sensitive.

#### 4.2.2. Contribution of Each Surveillance System’s Component

Despite optimistic assumptions about the screening of exchanged animals, this component had no significant part in the detection of TB. Therefore, this area-wide approach seems to confirm that stopping the screening of exchanged animals would have a negligible impact on the effectiveness of surveillance. Since this component is quite restrictive for field actors and of very low effectiveness, its interruption seems logical, especially since currently screening for animals exchanged from “at risk” herds is a priori very little applied in the field.

In heavily infected areas, periodic screening was the component that mainly ensured a high probability of detecting an infected herd (Figure 5), highlighting the importance of periodic screening in TB control. 

In the TB-free areas, the probability of detecting infection at a prevalence of 0.1% was quite high in areas where herds had a high probability of being linked to an outbreak, like in the Western areas (Figure 3). In these areas, the good overall sensitivity of the surveillance system was ensured in the model by tracing-on investigations alone since periodic screening was stopped there. Thus, we have shown that it is possible to achieve effective surveillance through this targeted surveillance component, which underlines the importance for areas to implement these tracing-on investigations in order to detect outbreaks before late detection at the slaughterhouse.

Upstream and neighbourhood links were of very little relevance to the effectiveness of the system, but their presence was probably underestimated and/or taken into account in the periodic screening (see Section 4.1).

Unfortunately, the effectiveness of zoning-based screening could not be estimated in this work due to the lack of information on zoning and on the probability of infection of a herd in a zone compared with the rest of the area. However, given the importance of local contamination in the maintenance of TB in France [22], this risk-based periodic screening modality seems to be the best way to further improve the sensitivity of ante-mortem surveillance in infected areas.

## 5. Conclusions

The ante-mortem surveillance system was found to have a satisfactory total effectiveness (when accounting for all its components) in detecting infection in areas with high prevalence. In addition, in areas that are a priori TB-free but where animal trade is most at risk of introducing infection (high probability of their herds to have a downstream link with a TB outbreak), the tracing-on investigations allowed the detection of infection at the prevalence threshold of 0.1% with a high probability. This underlines the importance of carrying out these investigations seriously.

On the contrary, the screening of exchanged animals did not significantly improve the effectiveness of the surveillance, despite the overestimation of the effectiveness of this component in our model. Thus, stopping these screening seems reasonable. According to these results and to a favourable notice of the French Agency for Food, Environmental, and Occupational Health and Safety [23], for the French 2020–2021 TB surveillance campaign, screening of exchanged animals remains mandatory only for animals from herds classified “at risk” following a downstream epidemiological link with an outbreak.

## Figures and Tables

**Table 1:** Calculation of the relative risk of TB infection for each herd category (RRi) used in the scenario tree modeling ante-mortem TB surveillance on a herd.

Size	Turnover	Surveillance Group	RRi
Small	<40%	At risk	C_risky_
		Former outbreak > 3 years	C_risky_
		None	1
	≥40%	At risk	C_high turnover_ × C_risky_
		Former outbreak > 3 years	C_high turnover_ × C_risky_
		None	C_high turnover_
Big	<40%	At risk	C_big_ × C_risky_
		Former outbreak > 3 years	C_big_ × C_risky_
		None	C_big_
	≥40%	At risk	C_big_ × C_high turnover_ × C_risky_
		Former outbreak > 3 years	C_big_ × C_high turnover_ × C_risky_
		None	C_big_ × C_high turnover_

C_big_: coefficient modeled by an asymmetric normal law N_asym_ (mean = 2; CI95% min = 1.4; CI95% max = 3). C_high turnover_: coefficient modeled by an asymmetrical normal distribution N_asym_ (mean = 1.2; CI95% min = 1; CI95% max = 1.5). C_risky_: coefficient modeled by a Pert distribution law: Pert (min = 1, mean = 2, max = 10).

**Table 2:** Summary of over- and underestimated parameters.

Overestimated Parameters	Underestimated Parameters
+ probability for a herd to be “at risk” + for some areas, proportion of herds subject to periodic screening + probability for animals sold from “at risk” herds to be screened	- proportion of use of the “strict” protocol (particularly in the South-West) - probability for a herd to be an upstream or neighbourhood link

## Appendix A

**Table A1:** Details of the prevalence set in each area for the evaluation of the TB ante-mortem surveillance system.

Area’s Identification Number	Area’s Name	Number of Outbreaks between 2011 and 2016	Prevalence Used for Modeling
01	Ain	[0–5[	0.0001
02	Aisne	[0–5[	0.0001
03	Allier	[0–5[	0.0001
04	Alpes-de-Haute-Provence	[0–5[	0.0001
05	Hautes-Alpes	[0–5[	0.0001
06	Alpes-Maritimes	[0–5[	0.0001
07	Ardèche	[0–5[	0.0001
08	Ardennes	10 et plus	0.006
09	Ariège	[5–10[	0.0002
10	Aube	[0–5[	0.0001
11	Aude	[0–5[	0.0001
12	Aveyron	[0–5[	0.0001
13	Bouches-du-Rhône	[0–5[	0.0001
14	Calvados	[0–5[	0.0001
15	Cantal	[0–5[	0.0001
16	Charente	10 et plus	0.006
17	Charente-Maritime	[5–10[	0.0002
18	Cher	[0–5[	0.0001
19	Corrèze	[0–5[	0.0001
2A	Corse-du-Sud	[0–5[	0.0001
2B	Haute-Corse	10 et plus	0.006
21	Côte-d’Or	10 et plus	0.006
22	Cotes-d’Armor	[0–5[	0.0001
23	Creuse	[0–5[	0.0001
24	Dordogne	10 et plus	0.006
25	Doubs	[0–5[	0.0001
26	Drome	[0–5[	0.0001
27	Eure	[0–5[	0.0001
28	Eure-et-Loir	[0–5[	0.0001
29	Finistère	[0–5[	0.0001
30	Gard	[0–5[	0.0001
31	Haute-Garonne	[0–5[	0.0001
32	Gers	[5–10[	0.0002
33	Gironde	[0–5[	0.0001
34	Hérault	[5–10[	0.0002
35	Ille-et-Vilaine	[0–5[	0.0001
36	Indre	[0–5[	0.0001
37	Indre-et-Loire	[0–5[	0.0001
38	Isère	[0–5[	0.0001
39	Jura	[0–5[	0.0001
40	Landes	10 et plus	0.006
41	Loir-et-Cher	[0–5[	0.0001
42	Loire	[5–10[	0.0002
43	Haute-Loire	[0–5[	0.0001
44	Loire-Atlantique	[0–5[	0.0001
45	Loiret	[0–5[	0.0001
46	Lot	[0–5[	0.0001
47	Lot-et-Garonne	10 et plus	0.006
48	Lozère	[0–5[	0.0001
49	Maine-et-Loire	[0–5[	0.0001
50	Manche	[5–10[	0.0002
51	Marne	[0–5[	0.0001
52	Haute-Marne	[0–5[	0.0001
53	Mayenne	[5–10[	0.0002
54	Meurthe-et-Moselle	[0–5[	0.0001
55	Meuse	[0–5[	0.0001
56	Morbihan	[0–5[	0.0001
57	Moselle	[0–5[	0.0001
58	Nièvre	[0–5[	0.0001
59	Nord	[0–5[	0.0001
60	Oise	[0–5[	0.0001
61	Orne	[0–5[	0.0001
62	Pas-de-Calais	[0–5[	0.0001
63	Puy-de-Dôme	[0–5[	0.0001
64	Pyrénées-Atlantiques	10 et plus	0.006
65	Hautes-Pyrénées	[0–5[	0.0001
66	Pyrénées-Orientales	[0–5[	0.0001
67	Bas-Rhin	[0–5[	0.0001
68	Haut-Rhin	[0–5[	0.0001
69	Rhône + métropole de Lyon	[0–5[	0.0001
70	Haute-Saône	[0–5[	0.0001
71	Saône-et-Loire	[0–5[	0.0001
72	Sarthe	[0–5[	0.0001
73	Savoie	[0–5[	0.0001
74	Haute-Savoie	[0–5[	0.0001
75	Paris	[0–5[	0.0001
76	Seine-Maritime	[5–10[	0.0002
77	Seine-et-Marne	[0–5[	0.0001
78	Yvelines	[0–5[	0.0001
79	Deux-Sèvres	[5–10[	0.0002
80	Somme	[0–5[	0.0001
81	Tarn	[0–5[	0.0001
82	Tarn-et-Garonne	[0–5[	0.0001
83	Var	[0–5[	0.0001
84	Vaucluse	[0–5[	0.0001
85	Vendée	[0–5[	0.0001
86	Vienne	[0–5[	0.0001
87	Haute-Vienne	[5–10[	0.0002
88	Vosges	[0–5[	0.0001
89	Yonne	[0–5[	0.0001
90	Territoire de Belfort	[0–5[	0.0001
91	Essonne	[0–5[	0.0001
92	Hauts-de-Seine	[0–5[	0.0001
93	Seine-Saint-Denis	[0–5[	0.0001
94	Val-de-Marne	[0–5[	0.0001
95	Val-d’Oise	[0–5[	0.0001

### Appendix A.1. Calculations A1. Deduction of a Modeling of the Raw Sensitivity and Specificity of the ICCT from French Data

No information on the raw sensitivity and specificity of ICCT was available in published studies. Indeed, all available estimates of sensitivity and specificity of the ICCT have been made with a strict interpretation of the test results (i.e., considering doubtful results as positive) since this is how the test is used in the field. To estimate the raw sensitivity and specificity of the ICCT, we therefore used a French database containing details of the screening test results (ICCT1 and, when they had been carried out, IFNγ, ICCT2, and results of laboratory analyses following diagnostic slaughter) of 14,270 cattle. Among these animals, 61% came from an area under enhanced surveillance and 37% from the IFN gamma protocol (protocol set up to evaluate the sensitivity and specificity of the IFNγ test in the field). These data were collected between 2013 and 2015 (2% of records came from an outbreak). This database allowed us to estimate the proportion of doubtful results on ICCT1 among non-negative results (positive or doubtful), depending on the infectious status of the cattle (confirmed infected or not following diagnostic culling). Among the confirmed infected animals with a non-negative result on ICCT1, 42% had a doubtful result. Among the unconfirmed infected (i.e., presumptively free) animals that had non-negative results on ICCT1, 84% had doubtful results. By rounding these percentages to 40% and 85%, respectively, we modeled the raw sensitivity of ICCT1 according to Equation (A1) and its raw specificity according to Equations (A2) and (A3). In these equations, Se_ICCT_ and Sp_ICCT_ are, respectively, the sensitivity and specificity of the ICCT1 with a strict interpretation as described in the published studies and modeled by asymmetric normal distribution laws: respectively, N_asym_ (min = 0.43; mean = 0.74; max = 0.95) and N_asym_ (min = 0.80; mean = 0.99; max = 1), bounded between 0 and 1.

(A1) Raw SeICCT = p (ICCT + | infected) = SeICCT × 1 − 0.40= SeICCT × 0.60

(A2) $$\begin{array}{c} P\;(ICCT+\;|\;uninfected)=\;\left(1\;-\;SpICCT\right)\;\times \;\left(1\;-\;0.85\right)\\ \;=\;\left(1\;-\;SpICCT\right)\;\times \;0.15\;\Leftrightarrow \;1\;-\;Raw\;SpICCT \end{array}$$

(A3) Raw SpICCT = 1 − 1 − SpICCT × 0.15

### Appendix A.2. Calculations A2. Method for Estimating the Probabilities of Obtaining a Positive or Negative Final Result in a Herd for Each of the Regulatory Protocols for Periodic Screening, Conditional on the Results of the ICCT1

- **Results of the Investigation Using the “Strict” Protocol Conditional on Obtaining at Least One Positive ICCT1 in the Herd**

At each iteration of the model, the numbers of positive ICCT1 results on infected (nb_I_ICCT+) and non-infected (nb_NI_ICCT+) animals were calculated for each herd type from equations including herd size (N), intra-herd prevalence (pr_intra_), and raw ICCT sensitivity and specificity (raw Se_ICCT_ and raw Sp_ICCT_), as presented below.

According to Bayes’ theorem, the probability that an animal is infected (I) knowing that it has obtained a positive result on an ICCT (p (I | ICCT+)) verifies the equality in Equation (A4).

(A4) $$\begin{array}{ll} p\;(I\;|\;\mathrm{ICCT}\;+)\;= & (p\;(\mathrm{ICCT}+┤|I)\;\times \;p\;\left(I\right))\;/\;\left(p\;\left(\mathrm{ICCT}+\right)\right)\\ & =\;\left(\mathrm{Raw}\;\mathrm{SeICCT}*\mathrm{pr}\_\mathrm{intra}\right)\;/\;(p\;(\mathrm{ICCT}+\;┤|\;I)\;\times \;p\;\left(I\right)\\ & +\;p\;(\mathrm{ICCT}\;+\;┤|\;\mathrm{NI})\;\times \;p\;\left(\mathrm{NI}\right))\Rightarrow \;p\;(I\;|\;\mathrm{ICCT}\;+)\\ & =\;\left(\mathrm{Raw}\;\mathrm{SeICCT}\times \mathrm{pr}\_\mathrm{intra}\right)/(\mathrm{Raw}\;\mathrm{SeICCT}\times \mathrm{pr}\_\mathrm{intra}\;+\;(1\\ & -\;\mathrm{Raw}\;\mathrm{SpICCT})\;\times \;(1\;-\;\mathrm{pr}\_\mathrm{intra})) \end{array}$$

In addition, the probability of an animal being non-infected (NI) knowing that it has obtained a positive result on an ICCT verifies the equality in Equation (A5).

(A5) p (NI | ICCT +) = 1 – p (I | ICCT +)

The numbers of infected and non-infected animals with positive ICCT1 were calculated at each iteration of the model with Equations (A6) and (A7) for infected herds and (A8) for TB free herds. In these equations, we forced the detection of at least one animal with a positive ICCT1 result (underlined parts of the following equations). anxsup24m is the proportion of animals over 24 months of age in the herd and the ICCT rate is the proportion of animals intended to be tested for ICCT1 that are actually tested.

(A6) $$\begin{array}{cc} \mathrm{Nb}\_I\_\mathrm{ICCT} & +\;\mathrm{infected}\;\mathrm{herd}\;\\ & \;=\;p\;(I\;|\;\mathrm{ICCT}\;+)\;\times \;1\;+\;(N\;\times {\;\mathrm{pr}}_{\mathrm{intra}}\;\times \;\mathrm{anxsup}24m\;\\ & \;\times \;\mathrm{ICCT}\;\mathrm{rate}\;–\;p\;(I\;|\;\mathrm{ICCT}\;+)\;\times \;1)\;\times \;\mathrm{Raw}\;\mathrm{SeICCT}\\ & \;\times \;\mathrm{anxsup}24m\;\times \;\mathrm{ICCT}\;\mathrm{rate} \end{array}$$

(A7) $$\begin{array}{cc} \mathrm{Nb}\_\mathrm{NI}\_\mathrm{ICCT}\;+ & \mathrm{infected}\;\mathrm{herd}\;\\ & \;=\;p\;(\mathrm{NI}\;|\;\mathrm{ICCT}\;+)\;\times \;1\;+\;(N\;\times \;(1\;-{\;\mathrm{pr}}_{\mathrm{intra}})\;\times \;\mathrm{anxsup}24m\;\\ & \;\times \;\mathrm{ICCT}\;\mathrm{rate}\;–\;p\;(\mathrm{NI}\;|\;\mathrm{ICCT}\;+)\times \;1)\;\times \;\left(1\;-\;\mathrm{Raw}\;\mathrm{SpICCT}\right) \end{array}$$

(A8) $$\begin{array}{cc} \;\mathrm{Nb}\_\mathrm{NI}\_\mathrm{ICCT}\;+\; & \mathrm{uninfected}\;\mathrm{herd}\\ & \;=\;1\;+\;\left(N\;\times \;\mathrm{anxsup}24m\;\times \;\mathrm{ICCT}\;\mathrm{rate}\;-\;1\right)\;\times \;(1\\ & \;-{\;\mathrm{Raw}\;\mathrm{Sp}}_{\mathrm{ICCT}}) \end{array}$$

The sensitivity and specificity of the “strict” protocol conditional on obtaining at least one positive ICCT was then calculated at each iteration, based on the scenario tree previously constructed [4,6], in which the number of animals obtaining a positive ICCT was set according to the values previously calculated (Equations (A6)–(A8)).

- **Results of the “compliant slow path” and “compliant quick path” protocols conditional on obtaining at least one non-negative ICCT1 but no positive ICCT1**

The sensitivity and specificity of these two protocols conditional on obtaining at least one non-negative but no positive ICCT1 were calculated, at each iteration, from the corresponding scenario trees described in previous studies [4,6]. For these calculations, the sensitivity of the ICCT1 was replaced by the probability of an infected animal obtaining a doubtful result knowing that it did not obtain a positive result (p(ICCT doubtful | ICCT not positive)_infected animal_), calculated according to Equation (A9). The specificity of ICCT1 was replaced by the probability of an non-infected animal obtaining a negative result knowing that it has not obtained a positive result (p(ICCT negative | ICCT not positive)_uninfected_), calculated according to Equations (A10) and (A11).

(A9) $$\begin{array}{c} p\;(\mathrm{ICCT}\;\mathrm{doubtful}\;|\;\mathrm{ICCT}\;\mathrm{not}\;\mathrm{positive}{)}_{\mathrm{infected}\;\mathrm{animal}}\\ =\;\frac{p(\mathrm{ICCT}\;\mathrm{not}\;\mathrm{positive}\;|\;\mathrm{ICCT}\;\mathrm{doubtful})\times p\left(\mathrm{ICCT}\;\mathrm{doubtful}\right)}{p\left(\mathrm{ICCT}\;\mathrm{not}\;\mathrm{positive}\right)}\\ p\;(\mathrm{ICCT}\;\mathrm{doubtful}\;|\;\mathrm{ICCT}\;\mathrm{not}\;\mathrm{positive}){\;}_{\mathrm{infected}\;\mathrm{animal}}=\;\frac{1\;\times \left(\mathrm{SeICCT}\times 0.4\right)}{1-\mathrm{Raw}\;\mathrm{SeICCT}}\;=\;\frac{1\;\times \;\mathrm{SeICCT}\times 0.4}{1-\mathrm{SeICCT}\times 0.6} \end{array}$$

(A10) $$\begin{array}{c} p\;(\mathrm{ICCT}\;\mathrm{doubtful}\;|\;\mathrm{ICCT}\;\mathrm{not}\;\mathrm{positive}{)}_{\mathrm{uninfected}\;\mathrm{animal}}\\ \;=\;\frac{p(\mathrm{ICCT}\;\mathrm{not}\;\mathrm{positive}\;|\;\mathrm{ICCT}\;\mathrm{doubtful})\times p(\mathrm{ICCT}\;\mathrm{doubtful})}{p(\mathrm{ICCT}\;\mathrm{not}\;\mathrm{positive})} \end{array}$$

(A11) $$p\;(\mathrm{ICCT}\;\mathrm{negative}\;|\;\mathrm{ICCT}\;\mathrm{not}\;\mathrm{positive}{)}_{\mathrm{uninfected}\;\mathrm{animal}}\;=\;1\;–\;p\;(\mathrm{ICCT}\;\mathrm{doubtful}\;|\;\mathrm{ICCT}\;\mathrm{not}\;\mathrm{positive})$$
